# Pleiomorphism plurihormonal Pit-1-positive macroadenoma with central hyperthyroidism: a rare case report and literature review

**DOI:** 10.1186/s12902-022-01220-2

**Published:** 2022-12-21

**Authors:** Guiliang Peng, Chuanhong Guo, Yangfan Lv, Dandan Li, Ling Zhou, Rufei Shen, Yong Chen, Xin Zheng, Zheng Sun, Hongting Zheng, Min Long

**Affiliations:** 1 Department of Endocrinology, translational Research Key Laboratory for Diabetes, The Second Affiliated Hospital (Xinqiao Hospital) of Army Medical University, 183 Xinqiao Zhengjie, Shapingba District, Chongqing, 400037 People’s Republic of China; 2 Department of Pathology, The Second Affiliated Hospital (Xinqiao Hospital) of Army Medical University, Chongqing, People’s Republic of China; 3 National Drug Clinical Trial Institute, The Second Affiliated Hospital (Xinqiao Hospital) of Army Medical University, Chongqing, People’s Republic of China; 4 Department of Neurosurgery, The Second Affiliated Hospital (Xinqiao Hospital) of Army Medical University, Chongqing, People’s Republic of China; 5 Department of Medicine, Division of Diabetes, Endocrinology and Metabolism, Baylor College of Medicine, Houston, TX USA; 6 Department of Endocrinology, The First Affiliation Hospital (Southwest Hospital) of Army Medical University, 30 Gaotanyan Zhengjie, Shapingba, Chongqing, 400038 People’s Republic of China

**Keywords:** Growth hormone (GH), Immunohistochemistry, Plurihormonal pit-1-positive adenoma, Pituitary neuroendocrine tumor (PitNET), Thyroid-stimulating hormone (TSH)

## Abstract

**Background:**

Thyrotropin-secreting pituitary neuroendocrine tumors (PitNETs) are rare pituitary adenomas that are occasionally accompanied by hypersecretion of other anterior pituitary hormones, such as growth hormone (GH) and prolactin (PRL). The clinical, biochemical, and pathological characteristics may represent diverse circumstances.

**Case presentation:**

In this report, a 33-year-old female diagnosed with a TSH PitNET co-secreting GH presented no obvious clinical symptoms. The main characteristics were elevated thyroid-stimulating hormone (TSH), free tri-iodothyronine (FT3), and free thyroxine (FT4) levels accompanied by slightly elevated GH and insulin-like growth factor-1 (IGF-1) levels. Magnetic resonance imaging (MRI) detected a pituitary macroadenoma (18 × 16 × 16 mm) with cavernous sinus and suprasellar invasion. Immunohistochemistry revealed diffuse positivity for TSH, strong immunoreactivity for GH, and sporadic positivity for PRL. The electron microscope and double immunofluorescence staining confirmed a plurimorphous plurihormonal adenoma producing TSH, GH, and PRL. After preoperative somatostatin receptor ligand (SRL) treatment and transsphenoidal surgery, the patient achieved temporary clinical and biochemical remission. However, 3 months after surgery, the patient was suspected of having Hashimoto’s thyroiditis due to higher thyroglobulin antibody (TGAb), thyroid peroxidase antibody (TPOAb), and thyroid receptor antibody (TRAb) and an enlarged thyroid nodule. During follow-up, thyroid function and TSH slowly transformed from transient hyperthyroidism to hypothyroidism. They were maintained in the normal range by L-T4.

**Conclusion:**

In the TSH PitNET, the positive immunohistochemistry for TSH, GH, and PRL translated into hormonal overproduction with TSH and GH.

**Supplementary Information:**

The online version contains supplementary material available at 10.1186/s12902-022-01220-2.

## Highlights

1. Plurihormonal pit-1-positive adenomas producing TSH, GH, and PRL are rare.
2. Mild or atypical clinical symptoms of TSH PitNET tend to be misdiagnosed.
3. Positive immunohistochemistry for one or more anterior pituitary hormones does not necessarily translate into hormonal overproduction or typical clinical symptoms.

## Background

Thyrotropin-secreting pituitary neuroendocrine tumor (PitNET) with a prevalence of 2–3 cases per million is an infrequent cause of central hyperthyroidism and is estimated to be less than 3% of all PitNETs [1, 2]. The chief characteristics of TSH PitNETs are inappropriate thyroid-stimulating hormone (TSH) levels (normal or high) and elevated free thyroxine (FT4) and free tri-iodothyronine (FT3) levels. Some patients with a long history of thyroid dysfunction tend to be easily misdiagnosed with Graves’ disease. Pit-1 is a common critical terminal differentiation factor and co-secretes in thyrotrophs, somatotrophs, and lactotrophs. One-fourth of TSH PitNETs are plurihormonal adenomas of Pit-1 lineage with hypersecretion of other pituitary hormones, mainly growth hormone (GH) (18%) and prolactin (PRL) (9%) [2].

To our knowledge, the frequency of pituitary adenomas producing TSH, GH, and PRL is relatively rare; only a few cases have been reported [3–11]. According to the 2017 World Health Organization (WHO) classification of tumors of the pituitary gland [12], a completely new definition is the plurihormonal Pit-1-positive adenoma, previously called silent adenoma subtype 3. Most of these tumors are clinically silent and rarely function as adenomas with acromegaly, hyperthyroidism, or hyperprolactinemia [13]. Additionally, as they are atypical lesions on conventional histology and immunohistochemistry, a definitive diagnosis usually requires ultrastructural confirmation. To provide more information for the clinician, this review focuses on the clinical, pathological, and therapeutic findings of a plurihormonal Pit-1-positive macroadenoma presenting with central hyperthyroidism.

## Case presentation

A 35-year-old female was referred to our department in October 2021 because of thyroid dysfunction for 2 months. Her past and family histories were unremarkable. In the local hospital, thyroid function tests demonstrated high levels of free tri-iodothyronine (FT3) 10.18 pmol/L (3.1–6.89 pmol/L), free thyroxine (FT4) 25.8 pmol/L (11–22 pmol/L), and thyroid-stimulating hormone (TSH) 8.6 mU/L (0.27–4.2 mIU/L). Thyroid ultrasound revealed a solid, slightly hyperechoic nodule in the left lobe of the thyroid (9.6 × 7.8 × 10.1 mm). Based on this funding, the patient was diagnosed with Graves’ disease and given methimazole (MMI, 5 mg bid). After 20 days, her thyroid function test was rechecked: FT3 was 7.97 pmol/L, FT4 was 19.8 pmol/L, and TSH was 10.49 mU/L. A local hospital suspected the patient had TSHoma. Further inquiries did not find a family history of thyroid dysfunction.

On examination at our hospital, her body mass index (BMI) was 22.8 kg/m^2^, her blood pressure and heart rate were 116/79 mmHg and 105 bpm, respectively, and her body temperature was 37 °C. Physical examination showed a degree 2 enlarged thyroid gland with no oculopathy or symptoms of acromegaly (Supplementary Figure 1).

### Preoperative laboratory examination

**Fig. 1:**
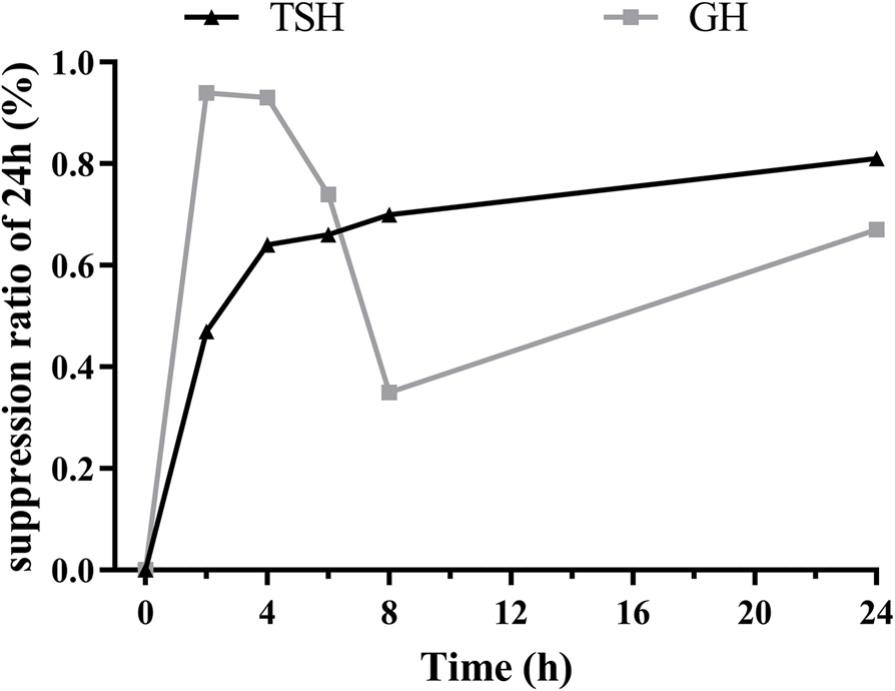
The suppression ratio of TSH and GH during 24 hours octreotide tests. The maximum suppression rate of GH was 94.4% at 2 hours; The maximum suppression ratio of TSH was 81.2% at 24 hours

Endocrine function tests showed an elevated TSH level of 12.26 mU/L with high levels of FT3 8.99 pmol/L and FT4 19.78 pmol/L. Sex hormone-binding globulin (SHBG) was 93.3 nmol/L (34.3–147 nmol/L). Thyroglobulin antibody (TGAb) was 271.70 IU/mL (0–115 IU/mL), thyroid peroxidase antibody (TPOAb) was 455.91 IU/mL (0–5.61 IU/mL), and thyroid receptor antibody (TRAb) was negative (Table 1). The α-subunit was not measured. After polyethylene glycol (PEG) precipitation, the TSH level was 9.1 to 3.48 mU/L, GH was 4.82 μg/L (0–8 μg/L), and IGF-1 insulin-like growth factor-1 (IGF-1) was 661.00 ng/mL (115–307 ng/mL, Table 1). An oral glucose tolerance test (OGTT) showed that the GH level decreased from 4.82 to 2.65 μg/L (Supplementary Figure 2). During a 24-h octreotide suppression test (0.1 mg subcutaneously every 4 hours during the first 12 h), TSH declined from 8.09 to 1.518 mIU/l, GH decreased from 4.31 to 0.24 mIU/l, and the suppression ratios of TSH and GH were 81.2 and 94.4%, respectively (Fig. 1). Other endocrine hormone levels were within the normal range: adrenocorticotropic hormone (ACTH) was 35.86 ng/mL (7.2–63.3 ng/L), cortisol was 304.2 nmol/L (66–579.4 nmol/L), PRL was 19.03 ng/mL, luteinizing hormone (LH) was 50.83 mIU/mL (follicular phase 239–66 mIU/mL; mid-cycle 9.06–72.24 mIU/mL; luteal phase 0.90–9.33 mIU/mL; postmenopausal 10.39–64.57 mIU/mL), follicular stimulating hormone (FSH) was 15.15 mIU/mL (follicular phase 3.03–8.08 mIU/mL; mid-cycle 2.55–16.69 mIU/mL; luteal phase 1.38–5.47 mIU/mL; postmenopausal 26.72–133.41 mIU/ml), estradiol was 238.00 pg/mL (follicular phase 21–251 pg/mL; mid-cycle 38–649 pg/mL; luteal phase 21–312 pg/mL; postmenopausal < 10–144 pg/mL), Testo (testosterone) was 1.29 nmol/L (0.38–1.97 nmol/L), and dehydroepiandrosterone (DHEA) was 121.3 μg/dL (139.7–484.4 μg/dL). Renal and liver function was within the normal range. Due to a drug deficiency in our center, the T3 test and thyrotropin-releasing hormone (TRH) stimulation were not applicable. Mutations in the TSH receptor and thyroxine receptor-β (THR-β) genes were not found.

**Table 1:** Pre and postoperative pituitary function surveys

	FT4 (pmol/L)	FT3 (pmol/L)	TSH (mIU/L)	TGAb (IU/mL)	TPOAb (IU/mL)	TG (ug/L)	TRAb **(**IU/L)	SHBG (nmol/L)	GH (ug/L)	IGF-1 (ng/mL)	PRL (ng/mL)
Pre-hospital											
D1 (**MMI**)	10.81	25.8	8.6	–	–	–	–	–	–	–	–
D23	7.97	19.98	10.49	–	–	–	–	–	–	–	–
D30	9.4	18.04	14.76	–	–	–	–	–	–	–	–
Pre-operation											
D1	8.99	19.78	12.268	271.7	455.91	4.02	1.13	93.3	4.82	661	19.03
D10 (**OCT**)	13.58	31.95	7.715	–	–	–	–	–	–	–	–
D12	7.78	25.95	1.208	–	–	–	–	–	1.15	354	9.61
D15	3.81	14.62	0.282	–	–	–	–	–	–	–	–
D18	3.33	13.03	0.299	–	–	–	–	–	–	–	–
Post-operation											
D1	2.58	14.87	0.044	–	–	–	–	–	2.1	268	19.26
D3	3.02	12.77	0.097	–	–	–	–	–	0.31	211	20.67
D7	2.75	9.9	0.299	–	–	–	–	–	0.16	274	24.06
1Mon	4.2	11.09	1.187	305.8	251.75	5.94	–	17.48	5.38	231	17.48
3Mon-a	8.15	22.85	0.187	285.6	735.26	20.35	2.93	69	0.65	299	12.82
3Mon-b	4.55	14.11	0.242	303.3	848.25	5.74	1.6	–	–	–	–
4Mon	2.92	6.41	6.772	330.6	> 1000	1.44	2.73	–	–	–	–
6mon (**L-T4**)	4.08	10.36	4.154	328.3	929.41	1.9	1.02	56	0.22	175	12.65

Abbreviation, *MMI* methimazole, *OCT* octreotide, *L-T*4 levothyroxine sodium tablets; hormone (reference range): *FT*3 free triiodothyronine (3.1–6.89 pmol/L), *FT*4 free thyroxine (11–22 pmol/L), *GH* growth hormone (0–3μg/L), *IGF*-1 insulin-like growth factor-1 (115–307 ng/mL), *PRL* prolactin (5.18–26.53 ng/mL), *SHBG* sex hormone-binding globulin (34.3–147 nmol/L), *TSH* thyroid-stimulating hormone (0.27–4.2mIU/L), *TGAb* thyroglobulin antibody (0–115 IU/mL), *TPOAb* thyroid peroxidase antibody (0–5.61 IU/mL), *TRAb* thyrotropin receptor antibody (0–1.75 IU/L), *TG* thyroglobulin (3.5–77μg/L)

### Preoperative imaging examination

**Fig. 2:**
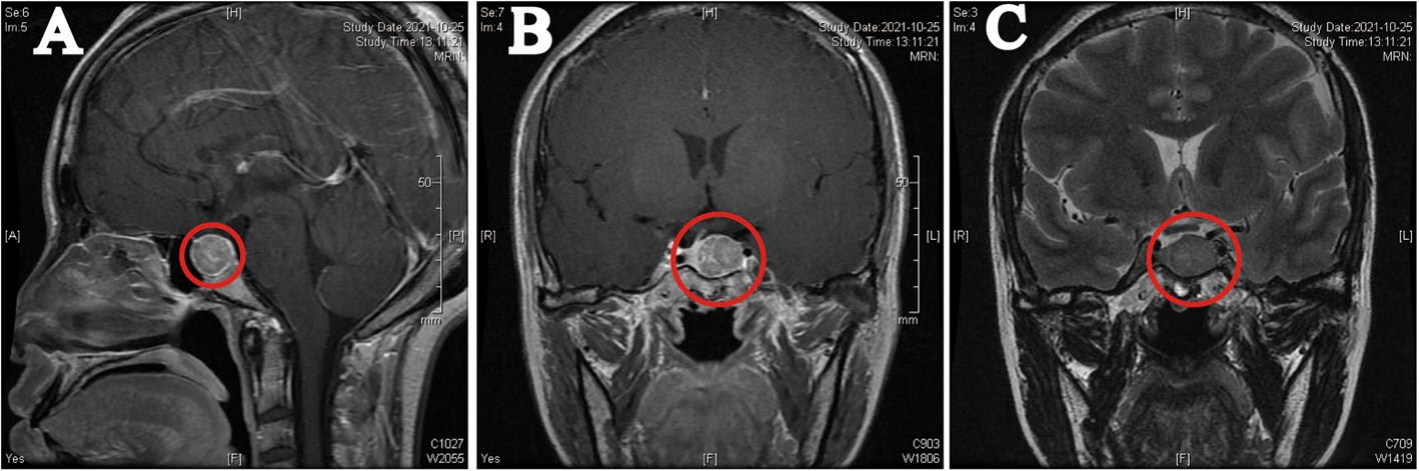
Brain magnetic resonance imaging. There was a pituitary macroadenoma (18 × 16 × 16 mm) with a quasi-circular equal signal shadow in the sellar region. **A**. gadolinium-enhanced sagittal T1WI, **B**. gadolinium-enhanced coronal T1WI, **C**. coronal T2WI

Her initial brain magnetic resonance imaging (MRI) revealed a quasi-circular equal signal shadow in the sellar region and a pituitary macroadenoma (18 × 16 × 16 mm) adjacent to the siphon of the left internal carotid artery (Fig. 2). The T2-weighted signal intensity was isointense. In addition, the Knosp and Hardy classifications of the pituitary tumor were grade 1 and 2, respectively. Additionally, the cavernous sinus invasion score (CSIS) was grade 1, the sphenoid sinus invasion score (SSIS) was grade 0, the suprasellar extension score (SSES) was grade 1, and the cumulative score was grade 2 [14]. The visual field revealed a visual field defect in the left eye. In thyroid echography, enlarged thyroid and hyperechoic nodules in the left lobe of the thyroid (11 × 8.3 × 11 mm, TI-RADS class 3) were observed. An ultrasound-guided fine-needle biopsy of the thyroid nodules showed benign lesions.

### Diagnosis and treatment

Based on these findings, this patient was finally diagnosed with TSH PitNET and acromegaly. After our multidisciplinary united team consultation, the patient was preoperatively treated with octreotide (OCT, 0.1 mg, s.c., tid). TSH, FT3, and FT4 levels decreased to the normal range in 5 days. Then, the pituitary mass was endoscopically removed via an endoscopic transsphenoidal resection. Surgical pathology confirmed a macroadenoma.

**Fig. 3:**
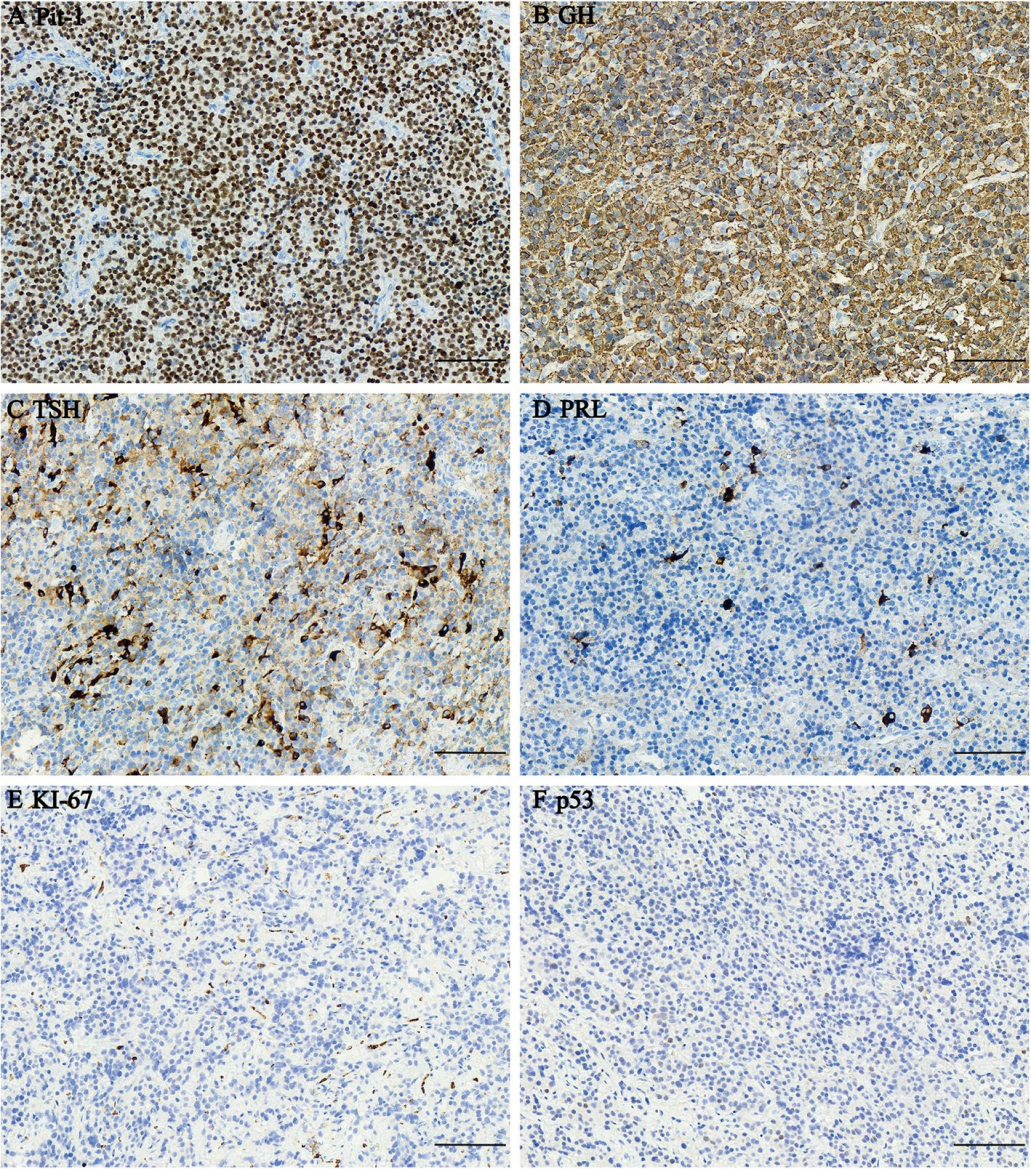
Histopathological findings: **a** IHC for Pit-1 revealed positive staining in adenoma cell nuclei. **b** IHC for GH. **c** IHC for TSH. **d** IHC for PRL. **e** IHC for KI-67. **f** IHC for p53. Original magnification × 200

**Fig. 4:**
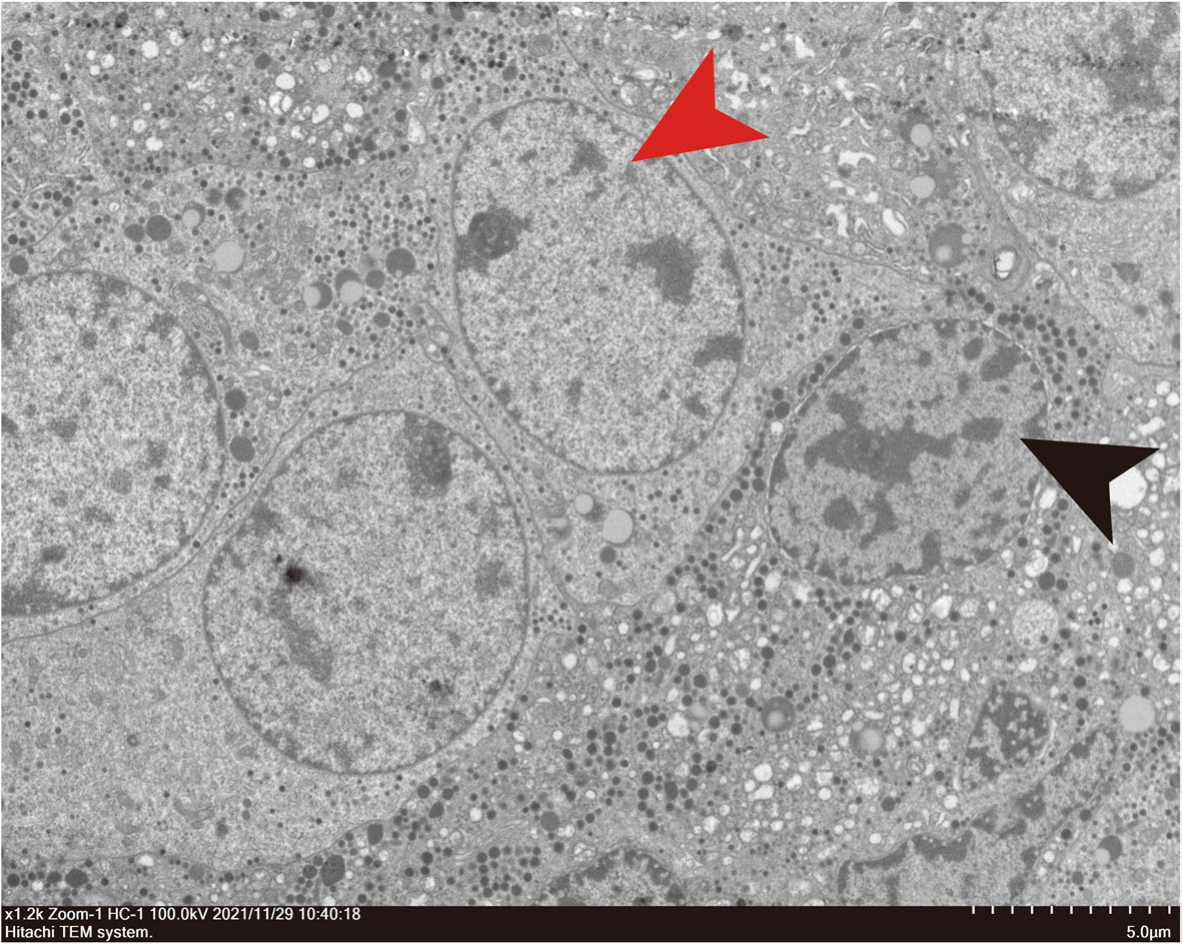
Electron microscopy revealed the adenoma cells were Plurimorphous. Ultrastructural examination identified that some adenoma cells were densely granulated, and secretory granules were spherical or ovoid and measured 300–450 nm (black arrow). The thyrotropin cells were a predominantly spherical or ovoid nucleus, Scattered lysosomes and mitochondria, prominent Golgi apparatus, and numerous secretory granules measuring 100 to 200 nm (red arrow)

Histologically, the tumor was composed of plurimorphic cells with a distinct cell border, abundant granulated cytoplasm, and round or oval nuclei. On light microscopy, histopathology revealed the plurihormonal adenoma with strong nuclear immunoreactivity with Pit-1 antibody (Fig. 3a). Immunohistochemistry revealed strong immunoreactivity for GH (Fig. 3b) and diffuse positivity for TSH (Fig. 3c) and PRL (Fig. 3d). The tumor had negative staining for ACTH, FSH, LH, and ER. In p53 staining, scattered p53-positive cells were observed (Fig. 3f). The Ki-67 index was < 1% (Fig. 3e). In GH and TSH immunohistochemical double staining, many GH cells and a minority of TSH cells were observed (Supplementary Figure 3B). Immunofluorescence double staining for GH and TSH positivity was found in different cell populations (Supplementary Figure 3A). In GH and PRL immunohistochemical and immunofluorescence double staining, GH was the most diffusely positive hormone, and PRL reactivity was scattered (Supplementary Figure 3C, D). Electron microscopy revealed that the tumor consisted of plurimorphous cells (Fig. 4). Some adenoma cells (black arrow) were densely granulated. Secretory granules were spherical or ovoid and measured 300–450 nm. Many adenoma cells contained elongated or geometrically shaped secretory granules, suggesting crystallization within their substance, a phenomenon seen in densely granulated somatotropic adenomas. The thyrotropin cells (red arrow) had a predominantly spherical or ovoid nucleus, scattered lysosomes and mitochondria, prominent Golgi apparatus, and numerous secretory granules measuring 100 to 200 nm. The cell membrane was peripherally clustered with numerous small secretory granules that outlined the cell boundary.

After surgery, TSH decreased slightly below the normal range (0.299 mIU/l), and FT3 and FT4 levels were normal at 2.75 pmol/L and 9.9 pmol/L, respectively. GH and IGF-1 levels were 0.16 μg/mL and 274 ng/mL 1 week after the surgery (Table 1). Except for high TGAb and TPOAb levels, TSH, FT3, FT4, GH, and IGF-1 levels decreased to the normal range 1 month after surgery. The OGTT test successfully suppressed GH below 0.4 μg/mL (basal GH, 7.62 μg/mL; maximal suppression, 0.4 μg/mL, Supplementary Figure 2). However, although no clinical symptoms of hyperthyroidism were observed in this patient, FT3 and FT4 levels increased, and TSH levels were slightly below the normal range 3 months after surgery (Table 1). Considering previously consistently elevated TGAb, TPOAb, and TRAb levels and an enlarged thyroid nodule, the patient was suspected of having Hashimoto’s thyroiditis. Interestingly, her thyroid function normalized over 10 days without medical treatment (Table 1). Four months after the surgery, her thyroid function showed increased TSH, TRAb, TPOAb, and TGAb levels and decreased FT4 levels. Hashimoto’s thyroiditis further destroyed the thyroid cells and led to hypothyroidism in this patient. Indeed, central hypothyroidism was also possible. Six months after the surgery, the patient was given levothyroxine sodium tablets (L-T4, 37.5 μg/day), and her FT4, TSH, and TRAb returned to the normal range (Table 1). The MRI revealed no particular abnormalities after the surgery. The patient achieved basal clinical and biochemical remission during follow-up.

## Discussion

This report described a case of plurihormonal pit-1-positive adenomas producing TSH, GH, and PRL. The clinical characteristics of the patient were elevated GH, IGF-1, and TSH levels without any obvious clinical symptoms. However, immunohistochemistry confirmed a plurimorphous plurihormonal macroadenoma producing GH, TSH, and PRL. A large study showed that 55% of somatotroph tumors produced other anterior pituitary hormones: PRL (77%), TSH (13%), and PRL-TSH (10%) [15]. Indeed, a meta-analysis revealed 61% of the TSHomas to be monohormonal and 39% to be plurihormonal co-secreting GH (57.5%) and PRL (41.4%) [16].

Several reports have shown plurihormonal tumors of the Pit-1 lineage to produce TSH, GH, and PRL [3–11]. Most of these plurihormonal TSHomas revealed hypersecretion of TSH and GH. The clinical symptoms were variable. Some presented with only hyperthyroidism [4, 8, 9] or without typical hypersecretion symptoms [3, 10]. Simultaneously, some patients showed acromegaly with hyperprolactinemia [7] or hyperthyroidism [5, 11]. Plurihormonal TSHomas with hypersecretion of GH revealed that acromegaly usually masks secondary hyperthyroidism [5]. A unique study revealed a plurihormonal TSHoma hypersecreting TSH, GH, and FSH [9]. Plurihormonal pituitary tumors may only present with a mass effect (headache and visual impairment) without other symptoms of a functioning pituitary tumor [6]. Although accompanied by hypersecretion of TSH and GH, this patient was dominated by features of thyroid dysfunction and was not suspected of acromegaly at presentation.

Immunocytochemical staining showed that this TSH PitNET was a plurihormonal tumor of Pit-1 lineage producing TSH, GH, and PRL. Concurrently, electron microscopy confirmed that TSH and GH were secreted by two different cells. Interestingly, although the pathology confirmed scattered PRL expression, the hypersecretion of PRL was not found in the blood. Previous cases also described plurimorphous plurihormonal adenomas producing TSH, GH, and PRL [17, 18]. However, in these reports, no fluorescent immunostaining studies had been administered to confirm the co-secretion of various hormones. In addition, Ozawa et al. reported that a plurihormonal adenoma co-secreting TSH and GH was ultrastructurally monomorphous in electron microscopy but immunohistochemically polymorphous [11]. Several reports confirmed that most plurihormonal TSHomas were monomorphous [4, 5, 8]. The plurihormonal Pit-1-positive tumors were characterized by a monomorphous population of cells expressing more than one hormone: TSH, GH, PRL, and α-subunit.

High-resolution pituitary MRI as a noninvasive technique can better characterize pituitary adenoma, predict postoperative pathology, and guide clinical treatment. The plurihormonal Pit-1-positive adenomas were highly aggressive and invasive, especially predominant suprasellar adenomas [19]. A previous case reported that a brain MRI in a pediatric patient revealed a giant plurihormonal pituitary adenoma with highly suprasellar extension, sphenoid, and bilateral cavernous sinus invasion (Knosp score 3) [5]. Differently, the tumor in our patient was typically macroadenoma with slightly cavernous and sphenoid sinus invasion (Knosp score 1). Additionally, T2 intensity indicated a significant relationship between histological subtype and the therapeutic effect of somatostatin receptor ligands (SRLs). T2 hyperintense GH-secreting adenomas were similar to sparsely granulated adenomas accompanied by typically higher cavernous sinus invasion and had a blunted response to first-generation SRLs [20]. Recently, studies reported that T2-signal intensity might change during SRL treatment, especially second-generation SRLs [21, 22]. In our case, immunohistochemistry demonstrated that GH cells had the characteristics of dense granules in electron microscopy. This tumor was isointense on T2-weighted magnetic imaging. The patient presented with hyperthyroidism and acromegaly received octreotide treatment, and obtained apparent clinical and biochemical control.

This case report had some limitations. First, the follow-up of this patient did not exceed 1 year. Second, this case performed no further colloidal gold double staining under the electron microscope. Third, the lack of somatostatin receptors 2 and 5 stainings could not help guide and evaluate the efficacy of SRLs.

## Conclusions

This case presented atypical clinical symptoms and hypersecretion of TSH and GH with normal PRL levels. However, the histology and ultrastructure proved that this pituitary adenoma had three distinct cells producing TSH, GH, and PRL. Concurrently, due to the combination of TSH PitNET and Hashimoto’s thyroiditis in this patient, dynamic testing of thyroid function (TSH, FT3, and FT4) and corresponding antibodies (TRAb, TPOAb, TG, and TGAb) helped to diagnose and identify the cause of thyroid function variations and guided follow-up treatment. Preoperative octreotide therapy combined with transsphenoidal surgery as the treatment for this patient ensured the safety of the perioperative period and achieved an excellent therapeutic effect.

## Supplementary Information

**Additional file 1:** **Supplementary Figure 1.** the facial and hand features. A. Facial characteristics revealed no enlargement of the nose and lips. B. the hand of control (left), the hand of the patient (right)

**Additional file 2:** **Supplementary Figure 2.** The dynamic curve of pre-op GH levels and post-op GH levels in 180 min-OGTT. The pre-op GH levels decreased from 4.82 to 2.65μg/mL; The post-op GH levels were successfully suppressed from 7.62to 0.4μg/mL (basal GH, 7.62μg/mL; maximal suppression, 0.4μg/mL)

**Additional file 3:** **Supplementary Figure 3.** Double immunohistochemistry and immunofluorescence staining of a Plurimorphous plurihormonal Pit-1 positive adenoma. A. TSH and GH double immunofluorescence staining, anti-GH is stained with Cy3 (red), anti-TSH with Actin-Tracker Green-488 (green), and nuclei with DAPI (blue). Original magnification × 630. B. TSH and GH double immunohistochemistry staining, GH is marked yellow, TSH is marked red, original magnification × 200. C. GH and PRL double immunofluorescence staining, anti-GH is stained with Cy3 (red), anti-PRL with Actin-Tracker Green-488 (green), and nuclei with DAPI (blue). Original magnification × 630. D. GH and PRL double immunohistochemistry staining, GH is marked yellow, PRL is marked red, original magnification × 200

## Data Availability

The datasets used and/or analyzed during the current study are available from the corresponding author upon reasonable request.

## Abbreviations

ACTH: Adrenocorticotropic hormone
CSIS: Cavernous sinus invasion score
DHEA: Dehydroepiandrosterone
FT3: Free tri-iodothyronine
FT4: Free thyroxine
FSH: Follicular stimulating hormone
GH: Growth hormone
IGF-1: Insulin-like growth factor-1
LH: Luteinizing hormone
MRI: Magnetic resonance imaging
MMI: Methimazole
OGTT: Oral glucose tolerance test
OCT: Octreotide
PRL: Prolactin
PEG: Polyethylene glycol
PitNETs: Pituitary neuroendocrine tumors
SRLs: Somatostatin Receptor ligands
SSES: Suprasellar extension score
SSIS: Sphenoid sinus invasion score
SHBG: Sex hormone-binding globulin
TSH: ThyrotropinTGAb: Thyroglobulin antibody
TPOAb: Thyroid peroxidase antibody
TRAb: Thyroid receptor antibody
Testo: Testosterone
