# Supplementary figures and images for: Pleiomorphism plurihormonal Pit-1-positive macroadenoma with central hyperthyroidism: a rare case report and literature review
Source: BMC Endocr Disord. 2022 Dec 21;22:325. doi: 10.1186/s12902-022-01220-2 (PMC9769035; doi:10.1186/s12902-022-01220-2)

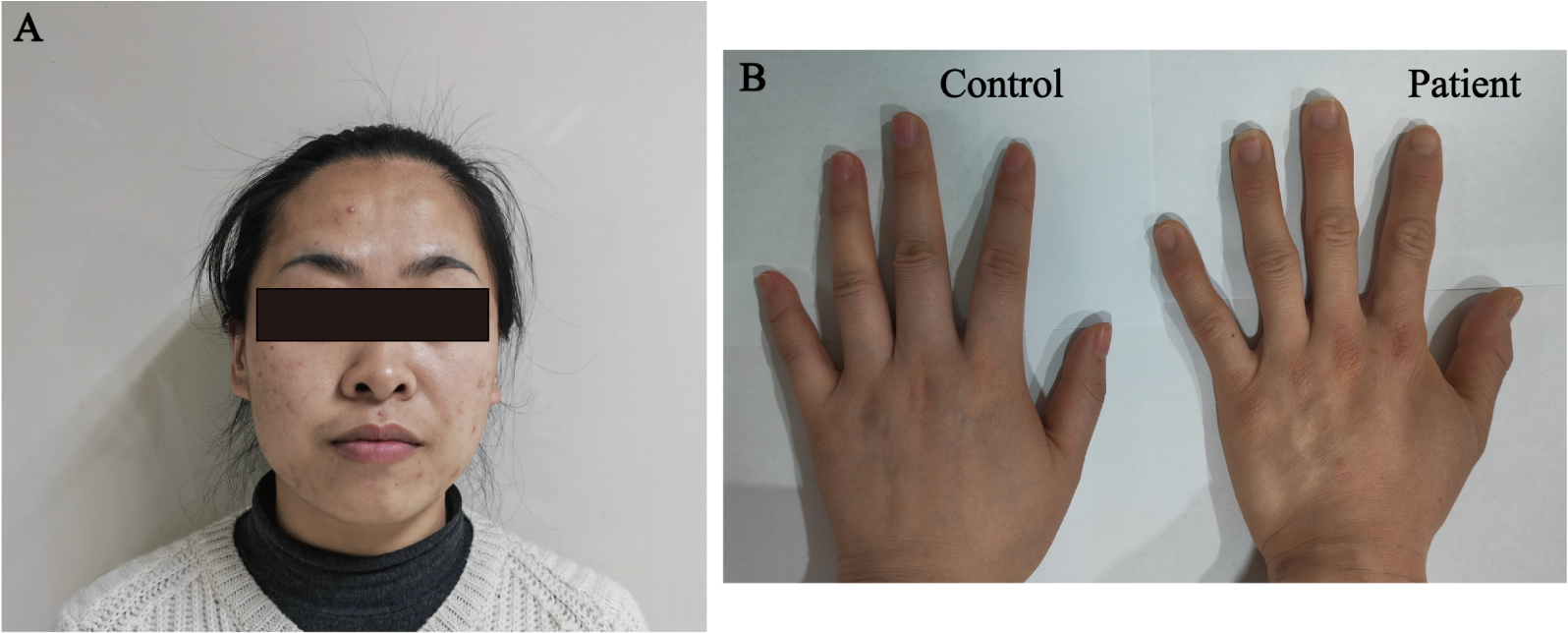

Supplement: Supplementary file 1 — Additional file 1: Supplementary Figure 1. the facial and hand features. A. Facial characteristics revealed no enlargement of the nose and lips. B. the hand of control (left), the hand of the patient (right) [file 12902_2022_1220_MOESM1_ESM.tif]

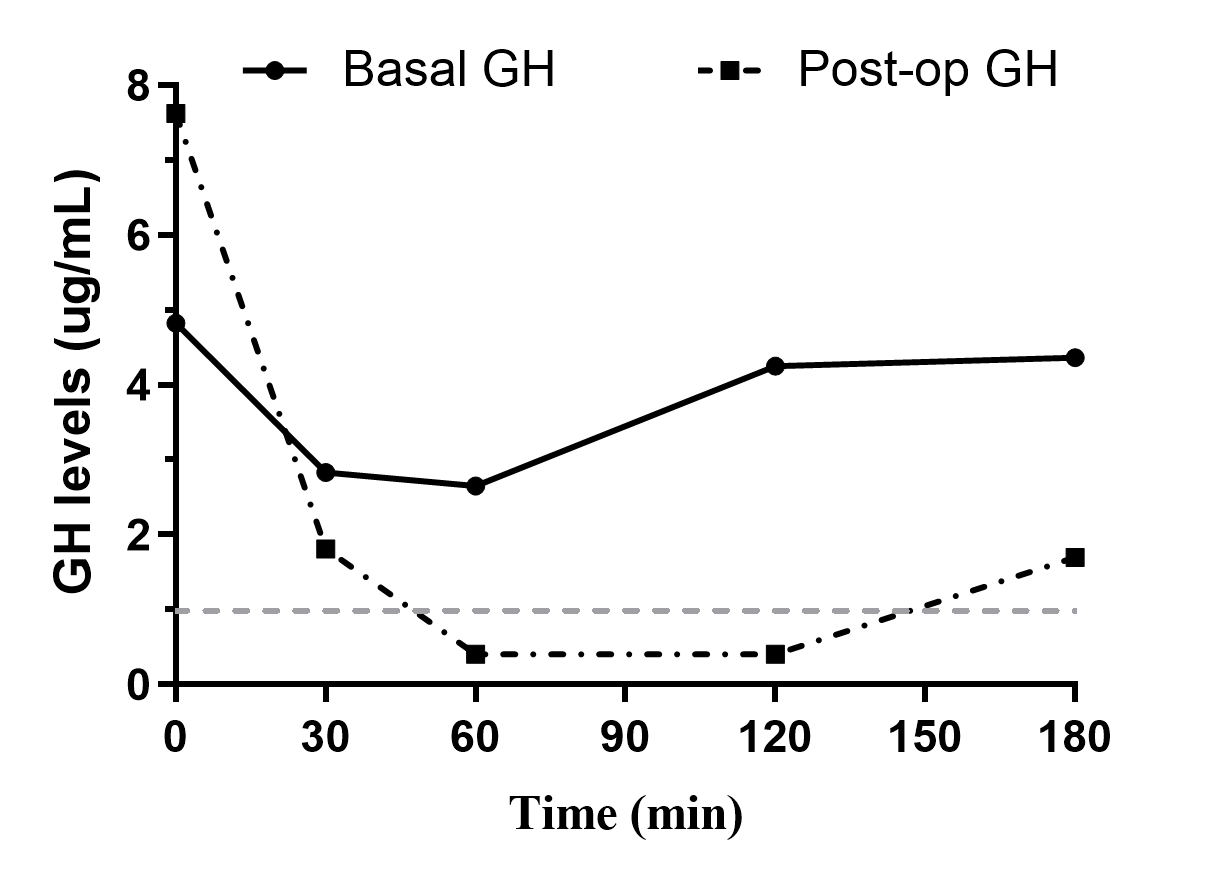

Supplement: Supplementary file 2 — Additional file 2: Supplementary Figure 2. The dynamic curve of pre-op GH levels and post-op GH levels in 180 min-OGTT. The pre-op GH levels decreased from 4.82 to 2.65μg/mL; The post-op GH levels were successfully suppressed from 7.62to 0.4μg/mL (basal GH, 7.62μg/mL; maximal suppression, 0.4μg/mL) [file 12902_2022_1220_MOESM2_ESM.tif]

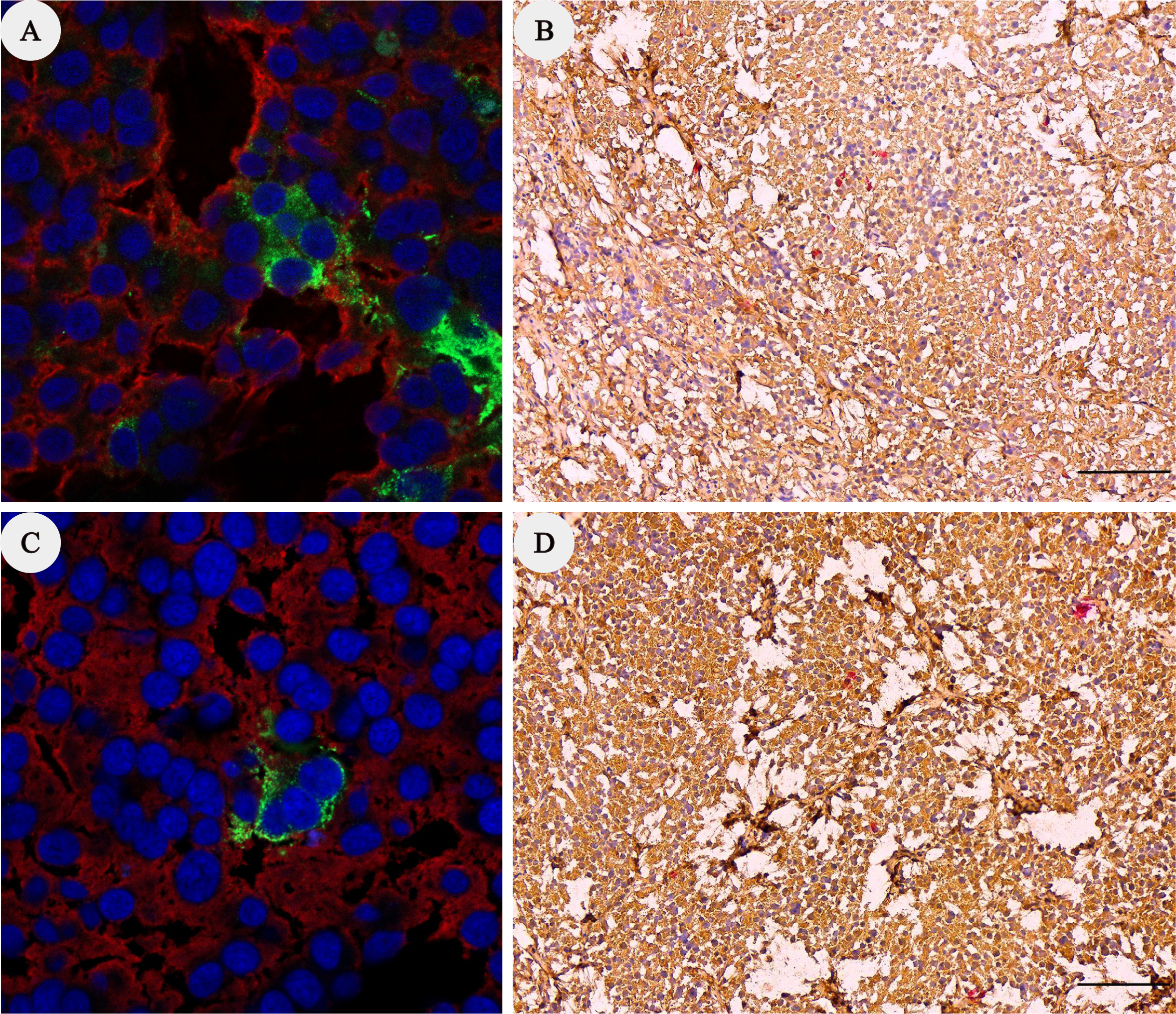

Supplement: Supplementary file 3 — Additional file 3: Supplementary Figure 3. Double immunohistochemistry and immunofluorescence staining of a Plurimorphous plurihormonal Pit-1 positive adenoma. A. TSH and GH double immunofluorescence staining, anti-GH is stained with Cy3 (red), anti-TSH with Actin-Tracker Green-488 (green), and nuclei with DAPI (blue). Original magnification × 630. B. TSH and GH double immunohistochemistry staining, GH is marked yellow, TSH is marked red, original magnification × 200. C. GH and PRL double immunofluorescence staining, anti-GH is stained with Cy3 (red), anti-PRL with Actin-Tracker Green-488 (green), and nuclei with DAPI (blue). Original magnification × 630. D. GH and PRL double immunohistochemistry staining, GH is marked yellow, PRL is marked red, original magnification × 200 [file 12902_2022_1220_MOESM3_ESM.tif]
